# Predictors, patterns, and correlates of moderate-severe psychological distress among New York City College Students during Waves 2–4 of COVID-19

**DOI:** 10.1038/s41598-025-86364-6

**Published:** 2025-01-25

**Authors:** Craig J. Heck, Deborah A. Theodore, April Autry, Brit Sovic, Cynthia Yang, Sarah Ann Anderson-Burnett, Caroline Ray, Eloise Austin, Joshua Rotbert, Jason Zucker, Marina Catallozzi, Magdalena E. Sobieszczyk, Delivette Castor

**Affiliations:** 1 Division of Infectious Diseases, Department of Medicine, Columbia University Irving Medical Center, 622 West 168th Street, Ste. 876, New York, NY 10032 USA; 2 Department of Epidemiology, Columbia University Mailman School of Public Health, New York, NY USA; 3 Barnard College, Health & Wellness, New York, NY USA; 4 Division of Child and Adolescent Health, Department of Pediatrics, Columbia University Irving Medical Center, New York, NY USA; 5 Heilbrunn Department of Population and Family Health, Columbia University Mailman School of Public Health, New York, NY USA

**Keywords:** Adolescents and young adults, Youth, Mental health, Longitudinal analysis, College health, Pandemic preparedness, Psychology, Risk factors

## Abstract

The COVID-19 pandemic may have exacerbated mental health conditions by introducing and/or modifying stressors, particularly in university populations. We examined longitudinal patterns, time-varying predictors, and contemporaneous correlates of moderate-severe psychological distress (MS-PD) among college students. During 2020–2021, participants completed self-administered questionnaires quarterly (T1 = 562, T2 = 334, T3 = 221, and T4 = 169). MS-PD reflected Kessler-6 scores ≥ 8. At T1 (baseline), most participants were cisgender women [96% vs. 4% transgender/gender non-conforming (TGNC)]. MS-PD prevalence was over 50% at all timepoints. MS-PD predictors included low self-rated health and perceptions of local pandemic control, verbal/physical violence experience, food insecurity, cohabitation dynamics, geographic location, and loneliness. Unique MS-PD correlates encompassed drug use and TGNC identity. Trajectories comprised Persistently (40%), Highly (24% MS-PD twice/thrice), Minimally (15% MS-PD once), and Never (21%) Distressed. Persistently Distressed students had low social support and self-rated health; high food insecurity, drug use, physical/verbal violence experience, need-based financial aid, and TGNC representation; and fluctuating self-rated health amid increasing COVID-19 symptomatology. In this sample, MS-PD prevalence was high, persistent, and associated with financial, behavioral, structural, experiential, and intra- and inter-personal factors. Given its complexity, improving and preserving college students’ mental health necessitates comprehensive, multi-component activities to change adjustable stressors while attenuating the adverse effects of immutable influences.

## Introduction

The United States is in the midst of a youth mental health crisis^1^. Between 2020–2021, > 60% of college students participating in the Healthy Minds survey were classified as having at least one mental health issue^2^. Since the Fall of 2020, ≥ 70% of undergraduates in the National College Health Assessment experienced psychological distress (PD), defined by the American Psychological Association as “painful mental and physical symptoms that are associated with normal fluctuations of mood”^3–9^. In some people, PD may be indicative of major depressive disorder, anxiety disorder, schizophrenia, somatization disorder, or a variety of other clinical conditions. In this unique population, negative mental health outcomes are consistently higher among cisgender women than men^10^. Individuals from gender minority groups also consistently report poorer mental health outcomes than their cisgender counterparts^11^.

Adverse mental health outcomes were increasing before the COVID-19 pandemic. Between 2009 and 2019, major depressive episodes among adolescent girls approximately doubled^12^. From 2011 to 2019, persistent feelings of sadness/hopelessness increased from 36 to 47% among female high school students^13^. Undergraduate cisgender women displayed marked increases in depression, anxiety, self-injury, and suicidal ideation, planning, and attempts between 2007 and 2017/2018^14^.

The COVID-19 pandemic catalyzed further decline of young cisgender women’s mental health^15^. Our prior research suggests that during remote learning in Winter 2020/2021, 73% of undergraduate cisgender women enrolled at a New York City (NYC) college experienced moderate-severe psychological distress (MS-PD). MS-PD was associated with greater need-based financial aid, food insecurity, verbal/physical violence experience, loneliness, and low social support^16^. Comparatively, the pooled prevalence of PD during COVID-19 was 58% (51–65%) in medical school students across Africa, Asia, Australia, East Asia, Europe, and North and South America^17^. Corroborating our findings, international cohorts of university students have also identified social support, living situation, loneliness, food insecurity, and financial insecurity as key factors associated with PD during the pandemic^18–24^.

Evolution of environmental, circumstantial, behavioral, and interpersonal conditions over time can lead to changes in mental health^25,26^. The COVID-19 pandemic was similarly dynamic, ever-changing with scientific advances, governmental and institutional directives, pharmaceutical and non-pharmaceutical interventions, seasonal and variant-fueled waves, and regional variability in epidemiologic trends and pandemic control activities^27^. We aimed to understand determinants and trends of mental health outcomes among college students across quarterly follow-up periods, between December 2020/January 2021 (a time of increased countermeasures) and November/December 2021 (after social restriction policies had been progressively relaxed each quarter). To achieve this goal, we performed longitudinal analyses to elucidate predictors and trajectories of MS-PD among students enrolled at a NYC college.

## Methods

### Cohort design

We conducted a longitudinal study to observe the prospective effects of the COVID-19 pandemic on the health and wellness of students, faculty, and staff affiliated with a NYC residential college. In December 2020/January 2021 (T1 [COVID-19 Wave 2]), investigators disseminated emails with study details and enrollment links to all students, faculty, and staff with an institutional email address. Based on screening questions, participants were eligible if aged ≥ 18 years; self-identified as student, faculty, or staff member; and understood English. Eligible participants provided written informed consent. Enrolled participants answered sociodemographic and physical, social, and psychological well-being questions via web-based, self-administered questionnaires managed by Research Electronic Data Capture (REDCap)^28^. After T1, study staff re-distributed questionnaires every 3–4 months (T2: March/April 2021 [COVID-19 Tail-end Wave 2], T3: July/August 2021 [COVID-19 Wave 3], T4: November/December 2021 [COVID-19 Wave 4]).

### Analytic sample

We limited this analysis to students given our focus on youth. We restricted the trajectory analysis to respondents with complete PD data. We considered participants lost to follow-up (LTFU) once they (1) did not respond to a survey, (2) did not provide MS-PD data, or (3) self-identified as staff or faculty.

### Measures

We explored self-reported sociodemographic, behavioral, relational, COVID-19, and psychosocial factors in this analysis (Supplemental Material 1).

#### Primary/dependent variable

We quantified PD using the Kessler-6 and dichotomized the scale to identify individuals experiencing MS-PD (scores ≥ 8) versus not^29^.

#### Independent variables

For sociodemographic indicators, we primarily used standard National Institutes of Health questions to examine age, racial and ethnic identity, school year, gender identity (cisgender women vs. transgender or gender non-conforming [TGNC]), living situation and location, and need-based financial aid. Food insecurity was captured using two measures from the United States Department of Agriculture’s Food Security Survey Module (bought food did not last, could not afford balanced meals)^30^. For behavioral variables, we assessed alcohol consumption and drug use^31^. Relational factors spanned relationship status, verbal/physical violence experience from partners/cohabitants^32,33^, and recent physical/sexual contact. COVID-19-related factors comprised perception of local pandemic control, self-rated health, vaccine status, perceived risk of acquiring COVID-19 relative to peers, and cumulative prevalence of COVID-19 symptoms^31,34^. Psychosocial factors included loneliness, social support, and MS-PD (when reporting predictors and trajectory characteristics)^31^.

### Statistical analyses

We analyzed data using R (v4.4.0). For descriptive and tabular analyses, we conducted chi-square tests for categorical variables and Kruskal–Wallis rank sum tests for continuous/discrete variables.

#### Factors associated with MS-PD

We identified predictors of MS-PD by fitting Poisson regressions with robust standard errors to calculate risk ratios^35^. Since current MS-PD was highly predictive of future MS-PD and could affect other factors, all analyses controlled for current MS-PD status, including unadjusted models (i.e., minimally adjusted). We created adjusted models by fitting all measures with a minimally adjusted *p* ≤ 0.05 into the same model. When violence experience was eligible for adjustment, it was fitted into a separate model since its inclusion changes the underlying population to only those partnered/living with someone.

Using the same model-building approach as above, we estimated marginal effects for those with complete MS-PD data by fitting generalized estimating equations (GEE) models with an autoregressive correlation matrix.

For exploratory purposes, we supplemented predictive and GEE analyses with correlative analyses.

#### Trajectory analyses

In the trajectory analyses, we categorized individuals based on cumulative MS-PD reports and examined inter- and intra-trajectory differences.

#### LTFU analyses

To assess how attrition may have affected findings, we used a GEE model (autoregressive correlation matrix) to examine T1 characteristic differences in those lost and included across time. We also compared T1 factors between those with incomplete and complete MS-PD data (Traj).

### Ethics

All research reported in this article was performed in accordance with the Declaration of Helsinki. Columbia University’s Institutional Review Board oversaw and approved all study procedures and analyses (#AAAT3032). Respondents electronically provided written informed consent through REDCap.

## Results

### Sample

Everyone screened (N = 977) was eligible, with 75% (728/977) providing informed consent and 666 completing the T1 survey. After removing non-students (35 faculty, 69 staff) and those with missing PD data (n = 6), 556 students made up the T1 sample; over time, 334, 221, and 169 remained in the analytic sample at T2, T3, and T4, respectively (Supplemental Material 2).

### Sample characteristics

With a median age of 20, most students identified as White (63%) and non-Hispanic (86%; Table 1). At T1, most respondents were cisgender women (96%) living with family (55%) in the New York City metro area (52%), with low levels of need-based financial aid (44%) and high levels of social support (74%). Conversely, food insecurity (13% ran out of food, 18% could not afford balanced meals), alcohol (32%) and drug (22%) use, and relationships (30%) were less common.

**Table 1:** Descriptive statistics of the sample across time.

Characteristic	Time				*p* value
	1, N = 556	2, N = 334	3, N = 221	4, N = 169	
Age (Continuous)	20 (19–21)	20 (19–21)	20 (19–20)	20 (19–20)	0.241
Racial identity					0.784
White	63% (336/534)	63% (203/321)	63% (135/213)	65% (105/161)	
Asian/Asian American	20% (107/534)	19% (61/321)	22% (46/213)	22% (36/161)	
Other/Multiracial	17% (91/534)	18% (57/321)	15% (32/213)	12% (20/161)	
Hispanic ethnicity					0.913
No	86% (470/546)	87% (284/326)	88% (189/215)	87% (143/164)	
Yes	14% (76/546)	13% (42/326)	12% (26/215)	13% (21/164)	
School year					0.139
First-year	26% (145/554)	28% (94/333)	30% (67/221)	30% (51/169)	
Sophomore	22% (121/554)	20% (68/333)	21% (47/221)	23% (39/169)	
Junior	28% (153/554)	30% (99/333)	32% (71/221)	33% (56/169)	
Senior	24% (135/554)	22% (72/333)	16% (36/221)	14% (23/169)	
Gender identity					0.455
Cisgender woman	96% (535/556)	96% (319/334)	95% (209/221)	93% (158/169)	
Transgender/Gender non-conforming	4% (21/556)	4% (15/334)	5% (12/221)	7% (11/169)	
Lives with…					**< 0.001**
Family	55% (301/551)	20% (66/334)	51% (111/216)	7% (11/158)	
Friends/Roommate/Significant other	40% (220/551)	72% (242/334)	43% (92/216)	75% (119/158)	
Alone	5% (30/551)	8% (26/334)	6% (13/216)	18% (28/158)	
Location					**< 0.001**
On-campus housing	3% (14/554)	34% (112/334)	16% (36/220)	65% (108/166)	
Off-campus NYC metro area	52% (288/554)	49% (165/334)	43% (95/220)	28% (47/166)	
Outside NYC metro area	45% (252/554)	17% (57/334)	40% (89/220)	7% (11/166)	
Received need-based financial aid					0.933
No	56% (300/537)	58% (186/322)	58% (122/211)	56% (88/157)	
Yes	44% (237/537)	42% (136/322)	42% (89/211)	44% (69/157)	
Ran out of food & had no money to buy more					0.126
No	87% (483/554)	86% (284/332)	92% (202/220)	90% (149/166)	
Yes	13% (71/554)	14% (48/332)	8% (18/220)	10% (17/166)	
Couldn’t afford balanced meals					0.57
No	82% (451/552)	80% (268/333)	85% (187/220)	83% (137/165)	
Yes	18% (101/552)	20% (65/333)	15% (33/220)	17% (28/165)	
Frequency of alcohol use					0.224
Never/Rare	68% (379/556)	66% (219/333)	62% (138/221)	61% (102/168)	
Moderate/High	32% (177/556)	34% (114/333)	38% (83/221)	39% (66/168)	
Frequency of drug use					0.98
Never/Rare	78% (436/556)	78% (259/332)	79% (174/220)	77% (130/168)	
Monthly/Weekly/Daily	22% (120/556)	22% (73/332)	21% (46/220)	23% (38/168)	
Relationship status					0.728
Single	70% (389/556)	70% (233/333)	67% (147/220)	67% (112/168)	
In some form of relationship	30% (167/556)	30% (100/333)	33% (73/220)	33% (56/168)	
Experienced physical/verbal violence					**< 0.001**
No	49% (259/534)	70% (223/318)	69% (144/209)	86% (120/140)	
Yes	51% (275/534)	30% (95/318)	31% (65/209)	14% (20/140)	
Close physical/sexual contact with non-household member					**< 0.001**
No	68% (366/542)	62% (201/322)	42% (88/209)	40% (63/159)	
Yes	32% (176/542)	38% (121/322)	58% (121/209)	60% (96/159)	
Perception of local pandemic control					**< 0.001**
High	12% (68/552)	26% (88/333)	60% (133/220)	70% (116/166)	
Some	22% (124/552)	35% (116/333)	25% (54/220)	19% (32/166)	
Low	65% (360/552)	39% (129/333)	15% (33/220)	11% (18/166)	
Self-rated health status					**< 0.001**
Excellent	22% (122/555)	14% (46/334)	18% (40/221)	15% (25/168)	
Very Good	44% (246/555)	35% (118/334)	42% (92/221)	38% (64/168)	
Good	25% (137/555)	33% (110/334)	30% (67/221)	33% (55/168)	
Fair/Poor	9% (50/555)	18% (60/334)	10% (22/221)	14% (24/168)	
Vaccine status					
Fully vaccinated	^†^	12% (41/334)	98% (215/219)	99% (167/168)	
Single dose	^†^	20% (67/334)	0% (1/219)	1% (1/168)	
No vaccine	^†^	68% (226/334)	1% (3/219)	0% (0/168)	
Perceived risk of COVID-19					**0.006**
Lower risk than others like me	32% (180/555)	34% (115/334)	29% (63/221)	19% (32/168)	
Same risk as others like me	61% (338/555)	59% (197/334)	68% (150/221)	74% (125/168)	
Higher risk than others like me	7% (37/555)	7% (22/334)	4% (8/221)	7% (11/168)	
Ever had COVID-19 symptoms					**< 0.001**
No	80% (436/547)	73% (243/331)	71% (157/221)	60% (102/169)	
Yes	20% (111/547)	27% (88/331)	29% (64/221)	40% (67/169)	
Since 3 months ago, feel…					**< 0.001**
Less lonely	39% (216/552)	45% (150/333)	55% (122/220)	64% (106/166)	
Same	20% (111/552)	23% (75/333)	21% (46/220)	20% (33/166)	
Lonelier	41% (225/552)	32% (108/333)	24% (52/220)	16% (27/166)	
Had a social support network					0.08
Yes	74% (404/547)	75% (250/333)	78% (171/218)	82% (134/163)	
Don’t know	13% (69/547)	14% (48/333)	11% (24/218)	13% (21/163)	
No	14% (74/547)	11% (35/333)	11% (23/218)	5% (8/163)	
Psychological distress scale (range 0–24)	11 (7–14)	10 (6–14)	8 (5–12)	8 (5–12)	**< 0.001**
Reported moderate-severe psychological distress					**< 0.001**
No	26% (147/556)	34% (115/334)	46% (101/221)	44% (75/169)	
Yes	74% (409/556)	66% (219/334)	54% (120/221)	56% (94/169)	

Median (25%-75%); % (n/N).

^†^COVID-19 vaccines were not available until after T1.

Over time (T1 to T4), living arrangements changed with easing pandemic countermeasures and/or time of year: most lived in communal on- or off-campus environments with peers at T2 (relaxation of COVID-19 physical distancing) and T4 (continued relaxation of COVID-19 countermeasures and return to campus for Fall semester) and at home with family at T3 (summer break). These cycles were accompanied by decreases in violence experience (51% at T1 to 14% at T4) and increases in close physical/sexual contact (32% to 60%) and high perceived local pandemic control (12% to 70%). Excellent/very good self-rated health decreased over time (66% to 53%). Vaccination increased with expanding access and eligibility (0.0% to 99.4%). Shifts away from lower perceived COVID-19 risk (32% to 19%) coincided with increased COVID-19 symptoms (20% to 40%) and decreased loneliness (41% to 16%).

The PD scale (Supplemental Material 3) was reliable (Cronbach’s Alpha = 0.84). PD decreased over time, yet most respondents reported MS-PD across all time-points (74% to 56%). For TGNC-identifying students, median PD scores (T1: 15, T2: 15, T3: 11, T4: 12) and MS-PD classifications (T1: 91%, T2: 87%, T3: 75%, T4: 91%) were markedly higher than for the overall cohort.

### Factors associated with MS-PD

#### Predictive models

**Fig. 1:**
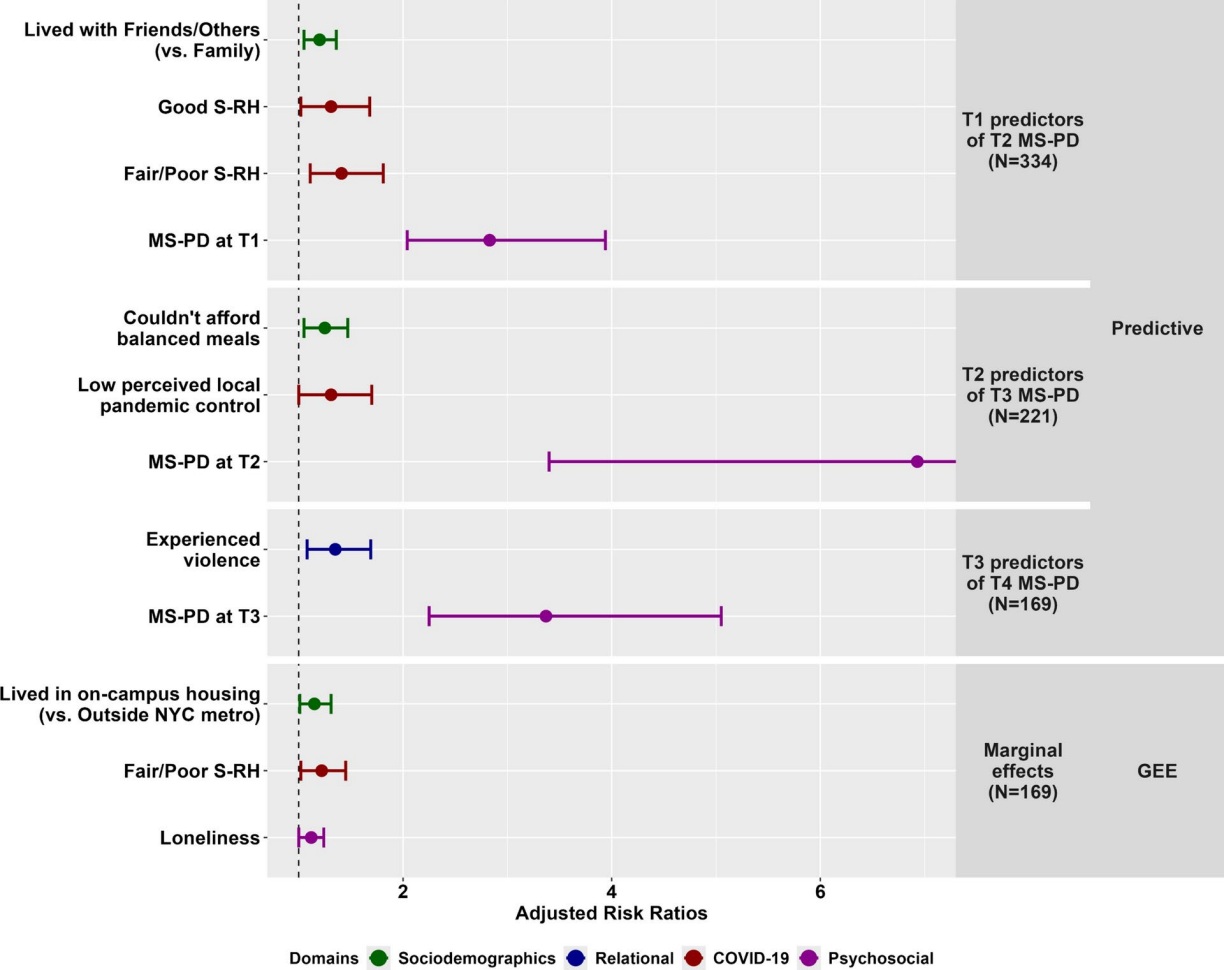
Predictors of Moderate-Severe Psychological Distress (MS-PD). S-RH = Self-Rated Health; The reference category for S-RH is “Excellent”. The reference category for perceived local pandemic control is “High”. The upper bound of the 95% confidence interval for MS-PD at T2 is 14.10. Because violence experience was the only other predictor (other than MS-PD) in the T3-T4 predictive analyses, these findings only apply to those partnered/living with someone at T3. Supplemental Materials 4 and 5 contain the full adjusted models, including factors with *p* > 0.05.

Figure 1 presents findings from the predictive models (Supplemental Material 4). In every analysis, current MS-PD predicted future MS-PD (T1: Adjusted Risk Ratio [ARR] = 2.83 [2.04–3.94], T2: ARR = 6.93 [3.40–14.10], T3: ARR = 3.37 [2.25–5.05]). The remaining predictors only arose at individual timepoints. At T1, living with friends/others (ARR = 1.20 [1.05–1.36], ref: Family) and low self-rated health (Good: ARR = 1.31 [1.02–1.68], Fair/Poor: ARR = 1.41 [1.11–1.81], ref: Excellent) predicted MS-PD at T2. Predictors of T3 MS-PD included inability to afford balanced meals (ARR = 1.25 [1.05–1.47]) and low perceived local pandemic control (ARR = 1.31 [1.00–1.70], ref: High) at T2. At T3, violence experience (ARR = 1.35 [1.08–1.69]) predicted MS-PD at T4.

#### GEE results

Figure 1 also highlights results from the GEE analysis (Supplemental Material 5). Like the predictive analyses, the marginal model found fair/poor self-rated health (ARR = 1.22 [1.02–1.45], ref: Excellent) increased MS-PD risk. Exclusive to the GEE model, living on-campus (ARR = 1.15 [1.01–1.31], ref: Outside NYC area) and loneliness (ARR = 1.12 [1.00–1.24]) increased risk of MS-PD.

#### Correlative findings

The correlative findings largely aligned with the predictive models (Supplemental Material 6). Low self-reported health was a positive correlate at every timepoint (T1: Very Good – Adjusted Prevalence Ratio [APR] = 1.20 [1.02–1.41], Good – APR = 1.27 [1.07–1.50], Fair/Poor – APR = 1.39 [1.17–1.65]; T2: Good – APR = 1.59 [1.11–2.26], Fair/Poor – APR = 1.93 [1.37–2.73]; T3: Good – APR = 2.09 [1.27–3.44], Fair/Poor – APR = 2.26 [1.33–3.82]; T4: Good – APR = 2.60 [1.25–5.42], Fair/Poor – APR = 3.53 [1.70–7.31]; ref: Excellent). Violence experience was also a positive correlate at T1 (APR = 1.16 [1.05–1.29]) and T2 (APR = 1.17 [1.00–1.37]). For inability to afford balanced meals, the T2 predictive relationship persisted into T3, when it was a positive correlate (APR = 1.33 [1.05–1.67]). Aligning with the GEE model, loneliness was associated with MS-PD at T1 (APR = 1.17 [1.05–1.32]) and T2 (APR = 1.20 [1.01–1.42]). Unique correlates included frequent drug use (T1: APR = 1.12 [1.01–1.24]) and TGNC identity (T4: APR = 1.63 [1.25–2.12]).

### Trajectory analysis

#### Sample characteristics

The trajectory sample (Supplemental Material 7) exhibited similar trends and PD scale reliability (Cronbach’s Alpha = 0.84) as the overall sample; greater alcohol use over time was the only notable differentiation from the overall sample.

#### Trajectory identification

Over time, 21%, 15%, 9%, 15%, and 40% of respondents reported zero, one, two, three, and four instances of MS-PD, respectively (Table 2). Respondents with zero and one MS-PD reports created the respective Never and Minimally Distressed trajectories; the latter primarily comprised MS-PD reports at T1 or T2. Respondents with two or three MS-PD reports were grouped into the Highly Distressed trajectory: most respondents exhibited MS-PD at T1-T2 and variable distress at T3 and T4. The Persistently Distressed trajectory included respondents reporting MS-PD at all time-points.

**Table 2:** Trajectories of moderate-severe psychological distress (N = 169).

Detailed trajectories	Number of reported moderate-severe psychological distress				
	Zero n = 36	One n = 25	Two n = 15	Three n = 25	Four n = 68
	Never Distressed Trajectory	Minimally Distressed Trajectory	Highly Distressed Trajectory		Persistently Distressed Trajectory
No/Low–No/Low–No/Low–No/Low	**100% (36)**	0% (0)	0% (0)	0% (0)	0% (0)
Mod-Sev–No/Low–No/Low–No/Low	0% (0)	**48% (12)**	0% (0)	0% (0)	0% (0)
No/Low–Mod-Sev–No/Low–No/Low	0% (0)	36% (9)	0% (0)	0% (0)	0% (0)
No/Low–No/Low–Mod-Sev–No/Low	0% (0)	4% (1)	0% (0)	0% (0)	0% (0)
No/Low–No/Low–No/Low–Mod-Sev	0% (0)	12% (3)	0% (0)	0% (0)	0% (0)
Mod-Sev–Mod-Sev–No/Low–No/Low	0% (0)	0% (0)	**40% (6)**	0% (0)	0% (0)
No/Low–No/Low–Mod-Sev–Mod-Sev	0% (0)	0% (0)	7% (1)	0% (0)	0% (0)
No/Low–Mod-Sev–No/Low–Mod-Sev	0% (0)	0% (0)	13% (2)	0% (0)	0% (0)
No/Low–Mod-Sev–Mod-Sev–No/Low	0% (0)	0% (0)	7% (1)	0% (0)	0% (0)
Mod-Sev–No/Low–No/Low–Mod-Sev	0% (0)	0% (0)	33% (5)	0% (0)	0% (0)
Mod-Sev–Mod-Sev–Mod-Sev–No/Low	0% (0)	0% (0)	0% (0)	**40% (10)**	0% (0)
Mod-Sev–Mod-Sev–No/Low–Mod-Sev	0% (0)	0% (0)	0% (0)	**40% (10)**	0% (0)
Mod-Sev–No/Low–Mod-Sev–Mod-Sev	0% (0)	0% (0)	0% (0)	4% (1)	0% (0)
No/Low–Mod-Sev–Mod-Sev–Mod-Sev	0% (0)	0% (0)	0% (0)	16% (4)	0% (0)
Mod-Sev–Mod-Sev–Mod-Sev–Mod-Sev	0% (0)	0% (0)	0% (0)	0% (0)	**100% (68)**

Mod-Sev = Moderate-Severe.

Bolded entries represent the most common detailed trajectory in each column.

#### Inter-trajectory differences

**Fig. 2:**
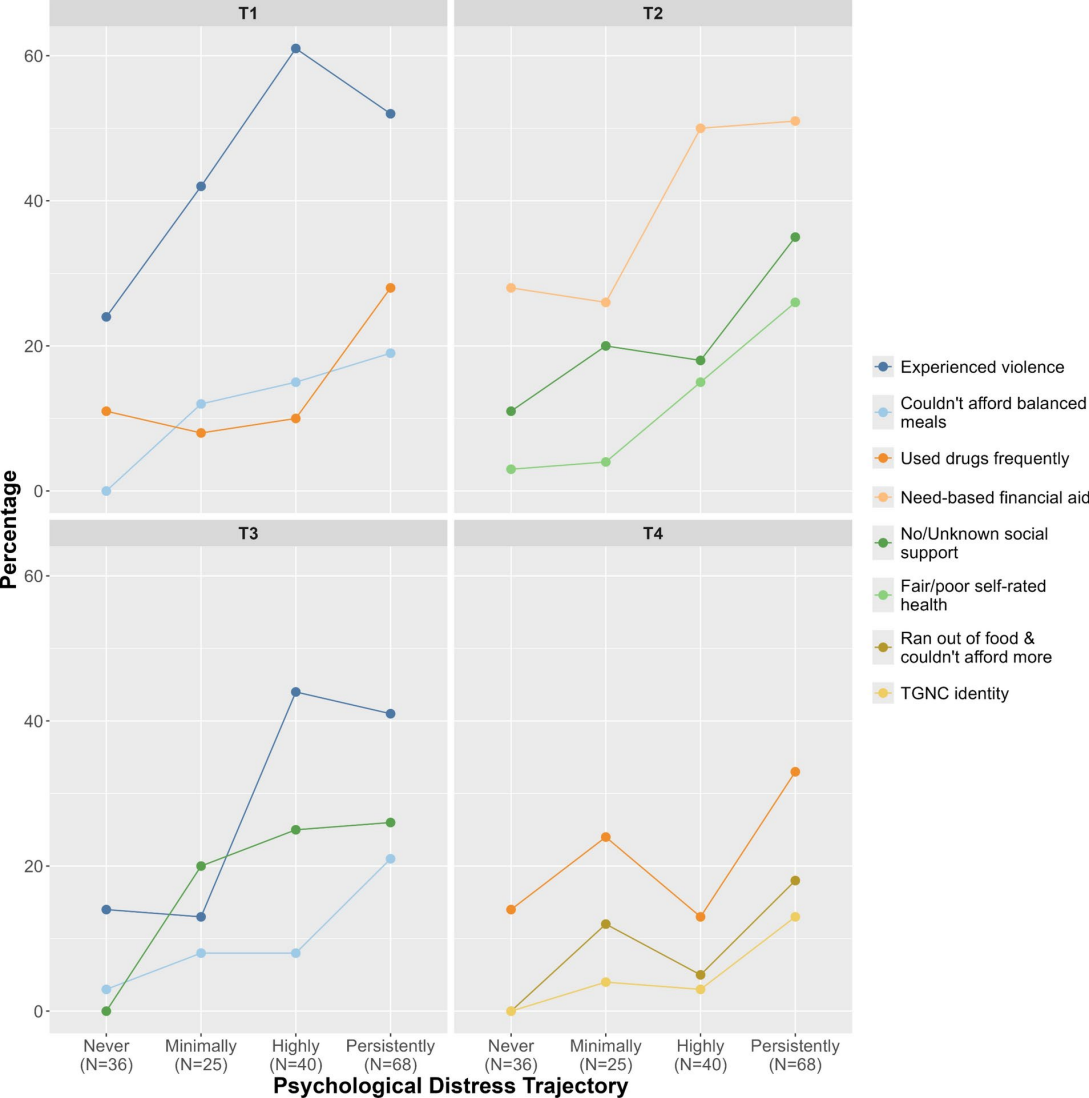
Inter-trajectory characteristic differences. TGNC = Transgender/Gender non-conforming; Supplemental Material 8 contains all analyses, including those with *p* > 0.05.

Figure 2 presents inter-trajectory differences across time (Supplemental Material 8). Relative to the other trajectories, the Persistently Distressed trajectory had high food insecurity (T1, T3, T4), drug use (T1, T4), violence experience (T1, T3), need-based financial aid (T2), and TGNC representation (T4) and low self-rated health (T2) and social support (T2, T3).

#### Intra-trajectory trends

**Fig. 3:**
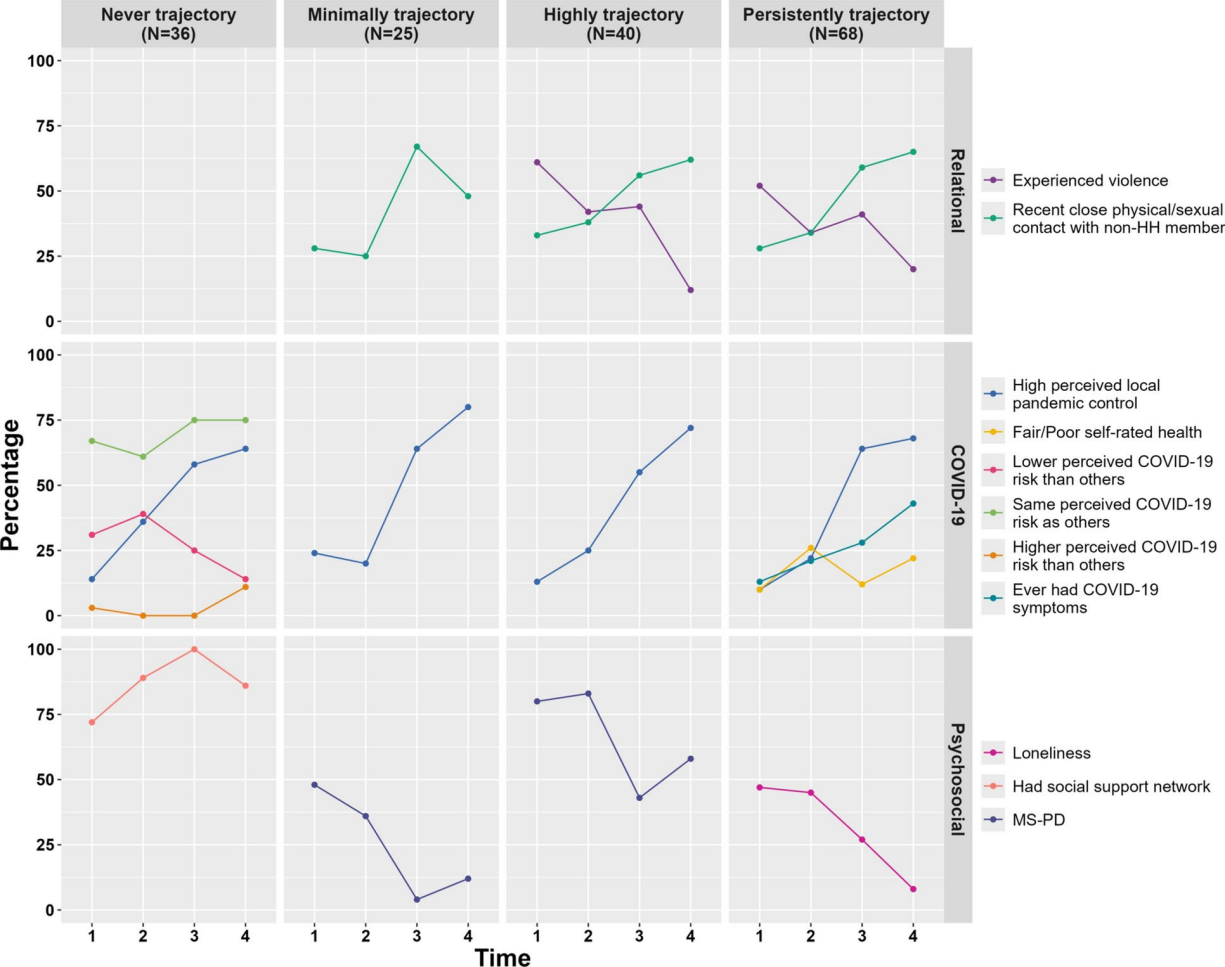
Intra-trajectory characteristic differences. HH = Household, MS-PD = Moderate-Severe Psychological Distress; Supplemental Material 9 contains all analyses, including those with *p* > 0.05.

Figure 3 showcases intra-trajectory changes over time (Supplemental Material 9). Violence experience decreased over time in the Highly and Persistently Distressed trajectories—with a slight uptick at T3. Close physical/sexual contact with non-household members increased among all trajectories except Never Distressed. Pandemic control perceptions improved among all trajectories, while Never Distressed trajectory members shifted from lower perceived risk to same or higher. In the Persistently Distressed trajectory, increased COVID-19 symptom experiences coincided with variable self-rated health and decreased loneliness. The Never Distressed trajectory had increasing social support. The highest MS-PD levels in the Minimally Distressed trajectory (T1, T2) were similar to the Highly Distressed trajectory’s lowest level (T3).

### LTFU analyses

Both models highlighted that those LTFU were more likely to be older (GEE: RR = 1.05 [1.01–1.10], Traj: PR = 1.05 [1.01–1.10]), Seniors (GEE: RR = 1.29 [1.12–1.49], Traj: PR = 1.28 [1.11–1.48], Ref: First-year), food insecure (Ran out of food—GEE: RR = 1.21 [1.06–1.37], Traj: PR = 1.21 [1.06–1.37]; No balanced meals—GEE: RR = 1.17 [1.04–1.33], Traj: PR = 1.16 [1.03–1.31]), and MS-PD (GEE: RR = 1.18 [1.03–1.36], Traj: PR = 1.19 [1.03–1.37]) at T1 (Supplemental Material 10).

## Discussion

Our analysis examined predictors, correlates, and trajectories of MS-PD among students enrolled at an NYC college. Over 12 months, we discovered MS-PD’s prevalence was consistently > 50%. MS-PD predictors encompassed current MS-PD, low self-rated health and perceptions of local pandemic control, food insecurity, physical/verbal violence experience, cohabitation dynamics, and geographic location. Unique MS-PD correlates included drug use, loneliness, and TGNC identity. Of those with complete data, four trajectories manifested: Persistently (40%), Highly (24% MS-PD twice/thrice), Minimally (15% MS-PD once), and Never (21%) Distressed. Persistently Distressed students had low self-rated health and social support; high food insecurity, drug use, physical/verbal violence experience, need-based financial aid, and TGNC representation; and over time, increasing COVID-19 symptomatology alongside varying self-rated health. College students’ PD is affected by intra-personal, behavioral, social, structural, circumstantial, and experiential factors; robust, multi-component efforts are necessary to intervene upon modifiable stimuli while mitigating adverse effects of immutable stressors.

Our measured PD levels exceeded national and international averages, a notable finding given the robust support services available during the study period. The cohort’s college offered 24-h full-spectrum medical and counseling services, including free therapy, groups, workshops, and medication to maintain emotional and mental health. Further, dedicated coordination and pandemic response teams managed COVID-19 testing, positive results, and communication about isolation; these teams also provided academic and food support to students under isolation precautions. Concerningly, we hypothesize PD levels were likely higher in other institutions without similarly robust support services. Retrospective mixed-methods analyses on the effect of specific support services on psychosocial outcomes during COVID-19 would help institutions prepare tailored interventions for future pandemics and/or shelter-in-place directives.

Low self-rated health emerged as an important MS-PD indicator: it predicted MS-PD in the T1-T2 analyses, emerged as a GEE factor, consistently coincided with MS-PD, and characterized the Persistently Distressed trajectory, at T2 and over time. Evidence from college students in France, Japan, and the United States support associative relationships between low self-rated health and poor mental health^36,37^. A longitudinal analysis with university students in Italy found similar life challenges—academic concerns, poor peer relationships and work-life balance, and worries about current and future financial strain—worsened mental and self-rated health, suggesting there may be similar synergies in our findings^38^. Since our self-rated health measure is framed generally, we cannot ascertain if students’ answers reflect awareness of their PD and links between mental and physical health. For example, increases in COVID-19 symptoms over time despite being fully vaccinated might have jointly affected self-rated health and MS-PD. More nuanced examination of self-rated health domains would clarify relationships between these factors.

Physical/verbal violence experience emerged as an MS-PD predictor and correlate, and it was high among the Highly and Persistently Distressed trajectories. Connections between college students’ violence experience and mental health outcomes are well documented, and our study further reinforces this relationship^39,40^. Violence was highest at T1 and T3, when most lived with family, suggesting that students may particularly benefit from directed resources while living at home. During COVID-19, familial and domestic violence increased due to shelter-in-place orders and environmental stimuli exacerbating underlying stressors, mental issues, and power differentials^41^. One potential mitigation tactic is financial and institutional support so students can voluntarily decide to remain on campus during academic breaks. Virtual services should also be available for remote learners, and emergency resources, like off-cycle campus returns, also warrant exploration.

Food insecurity was always higher among Highly or Persistently Distressed trajectories, except at T2, when it was supplanted by need-based financial aid. Food insecurity can negatively affect mental health outcomes directly and indirectly by adversely impacting mood, cognition, sociability, and sleep, and it has previously been shown to be associated with poor mental health outcomes among university students during the pandemic^22,42–45^. Food insecurity’s overlap with need-based financial aid suggests it is an apt proxy for financial insecurity^46,47^. Food insecure students might be unable to afford other essentials that promote positive mental health, like psychiatric services and gender-affirming care^48,49^. Notably, food insecurity at T2 predicted MS-PD at T3, but it was not a correlate until T3, potentially due to the mitigating effect of governmental stimulus checks distributed during T1 ($600) and T2 ($1400)^50^. Food pantries can attenuate food insecurity, but schools must stock them with healthy, nutritious options to avoid perpetuating malnutrition^51^. Anonymous food stipends might be an alternative strategy for students who are remote or do not access food pantries due to stigma or inconvenient locations^52^.

Although perceptions of local pandemic control improved overall, low perceived local pandemic control predicted MS-PD at T3. Perceptions of local pandemic control could have reflected local governmental policies, residents’ behaviors, COVID-19 morbidity and mortality rates, or a combination of these factors^53^. Living on-campus also increased risk of MS-PD in the GEE model, a finding possibly corresponding to international students, who likely remained on-campus during the entire study period due to travel restrictions^54,55^. During the study period, COVID-19 cases were high in the NYC metro area^56^. Living in this high-transmission environment likely made students concerned about potential COVID-19 exposures, elevating PD.

TGNC representation in our sample was approximately double the national average^4–9^. At T4, TGNC identity was correlated with MS-PD, and TGNC-identifying students had greater representation in the Persistently Distressed trajectory. These findings suggest MS-PD among cisgender respondents decreased enough to allow unmasking of TGNC-identifying students’ persistent MS-PD: the lowest median PD score among TGNC-identifying students corresponded to the highest median PD score in cisgender respondents. Colleges can mitigate PD through supportive policies and interventions to increase school safety, connectedness, and belonging^57^. Future research must include and engage TGNC-identifying students to better understand their unique circumstances, experiences, needs, and stressors.

The remaining stressors are likely interconnected: loneliness (T1, T2 correlate and GEE factor) and drug use (T1 correlate) coincided with remote learning, and the Persistently Distressed trajectory was typified by higher drug use (T1, T4) and low social support (T2, T3). Our findings align with previous studies that have identified low social support, drug use, and loneliness as factors associated with poor mental health outcomes among university students during the pandemic^18,19,23–25,58^. Creating local in-person and virtual social groups may support students during seasonal breaks or remote instruction and supplement counseling services. Future research should also explore if drug use exacerbated MS-PD or served as a coping mechanism.

Attrition may have affected our findings. Both LTFU analyses found inability to buy more food and afford balanced meals at T1 increased risk of LTFU, offering a potential reason for their null associations in the unadjusted or adjusted GEE findings. However, this point may also imply that the effects of unbalanced meals in the predictive (T2-T3) and correlative (T3) analyses are stronger than observed. Age and school year at T1 were significant factors in both LTFU models, but these findings are expected since most seniors graduated after T2. Graduates still had access to their institutional emails, but it is unlikely they checked it as frequently as current students. Finally, those with MS-PD at T1 were more likely to be lost, suggesting MS-PD may be higher in this cohort than measured, a concerning prospect since observed levels are already high.

Our analysis has some limitations. First, while others use a threshold of 5, we categorized MS-PD based on Kessler-6 scores ≥ 8, the same approach used by the scale’s creator^29^. We believed a conservative approach would reduce the risk of misclassifying non-PD individuals, which could potentially generate erroneous findings. Second, the survey did not measure sexual orientation, so we could not measure PD experiences among this sub-group. Third, although the survey was self-administered and participants were told all data would be deidentified, we cannot rule out social desirability bias affecting our conclusions. Fourth, we observed high levels of attrition among participants over time; thus, our findings should be interpreted as preliminary. Fifth, we could not examine international students’ unique experiences due to questionnaire limitations. Sixth, our findings may not apply to all college students given the preponderance of the sample was cisgender women.

## Conclusion

The COVID-19 pandemic has directly and indirectly exacerbated PD among college students by introducing or magnifying social, structural, intra-personal, and mental health vulnerabilities. In our sample, MS-PD was remarkably high and consistently associated with low self-rated health and perceptions of local pandemic control, physical/verbal violence experience, and food insecurity and—to a lesser propensity—drug use, loneliness, and TGNC identity. Persistently Distressed members, the most prevalent trajectory, were characterized by low self-rated health and social support; high food insecurity, physical/verbal violence experience, drug use, and TGNC representation; and variable self-rated health amid increasing COVID-19 symptomology. Higher education and public health institutions must ameliorate, nurture, and preserve college students’ mental health to foster positive health outcomes proximally and across the life course.

## Supplementary Information

Supplementary Information 1.

Supplementary Information 2.

Supplementary Information 3.

Supplementary Information 4.

Supplementary Information 5.

Supplementary Information 6.

Supplementary Information 7.

Supplementary Information 8.

Supplementary Information 9.

Supplementary Information 10.

## Data Availability

Due to the sensitive nature of these data and this population, the data are not publicly available. Requests to access the data used within this study should be directed to the corresponding author.
