# Supplementary material for: Predictors, patterns, and correlates of moderate-severe psychological distress among New York City College Students during Waves 2–4 of COVID-19
Source: Sci Rep. 2025 Jan 25;15:3206. doi: 10.1038/s41598-025-86364-6 (PMC11762720; doi:10.1038/s41598-025-86364-6)
Supplement: Supplementary file 1 — Supplementary Information 1. [file 41598_2025_86364_MOESM1_ESM.docx]

**Supplemental Material 1. Measure Definitions**

| **Variable** | **Type** | **Temporality** | **Definition** |
| --- | --- | --- | --- |
| ***Sociodemographic domain*** | | | |
| Age | Discrete | T1 only | Age in years |
| Racial identity | Polytomous | T1 only | Categorized as White vs. Asian/Asian American vs. Multiracial/Other   Multiracial/Other category contains respondents who identified as: more than one racial category (includes all American Indian/Alaskan Native and Native Hawaiian/Pacific Islander respondents), Black/African American (due to low representation), or Other (e.g., Middle Eastern, Jewish). |
| Hispanic ethnicity | Binary | T1 only | Coded as Hispanic vs. Not. |
| School year | Polytomous | T1 only | Matriculation stage upon entering the cohort: First-year vs. Sophomore vs. Junior vs. Senior |
| Gender identity | Binary | Contemporaneous | Represents self-identified Cisgender women vs. Transgender or Gender non-conforming students.   This variable was dichotomized due to low representation (<5%) of transgender and gender non-conforming respondents, inclusive of non-binary, transgender, gender non-conforming, questioning, agender, and demigender identities. |
| Living situation | Polytomous | Contemporaneous | With Family vs. With Friends, Roommates, Suitemates, or Significant Others vs. Alone. If a person reported living with both friends and family, we randomized them to either the friends or family category. |
| Location | Polytomous | Contemporaneous | Differentiated between people located On-campus vs. Off-campus in the New York City metro area vs. Outside the New York City metro area. |
| Need-based financial aid | Binary | Contemporaneous | Received need-based financial aid vs. Not. |
| Food not lasting | Binary | Contemporaneous | In the last 30 days, the food they bought often or sometimes (vs. never) didn’t last, and they didn’t have money to get more. |
| No balanced meals | Binary | Contemporaneous | In the last 30 days, they often or sometimes (vs. never) could not afford to eat balanced meals. |
| ***Behavioral domain*** | | | |
| Alcohol consumption | Binary | Contemporaneous | Alcohol consumption in the past 30 days, coded as low (never or <1-2 days a week), moderate (1-2 days a week), and high (3-4 days a week, daily or almost every day, more than once a day). The moderate and high categories were combined because the high category had low representation (i.e., <13%) at all time-points. |
| Drug use | Binary | Contemporaneous | Drug use in the past 30 days, coded as none/rare (never or less than once a month), moderate (1-3 days a month), and high (1-2 days a week, 3-4 days a week, daily or almost every day). We collapsed the moderate and high categories because each category had low representation (i.e., <13%) across all follow-up periods. |
| ***Relational domain*** | | | |
| Relationship status | Binary | Contemporaneous | In some form of relationship vs. Single. The relationship categorization includes those who are married; engaged; or in one, multiple, or complex relationships. Complex relationships encompass situationships, casual dating, exclusive and non-exclusive partnerships, and "it's complicated." |
| Experience of violence | Binary | Contemporaneous | In the past 30 days, a partner, spouse, or person they live with verbally (yelled at them or said things that made them feel bad about themselves, embarrassed in front of others, or frightened them) or physically (push, grab, hit, slap, kick, or throw things at them during an argument or because they were angry with them) assaulted them. Respondents were only asked these questions if they did not live alone or were in a relationship. |
| Close physical/sexual contract with non-household member | Binary | Contemporaneous | Kissed, hugged, or had any kind of sexual contact with at least 1 person outside their household in the last 7 days |
| ***COVID-19 domain*** | | | |
| Perception of pandemic control in their area | Polytomous | Contemporaneous | Assessment of COVID-19 pandemic being under control in the area they lived. Due to small cell sizes (<5% at two or more timepoints), response categories were collapsed into High (Very Much, Moderately), Some (Somewhat), and Low (Slightly, Not at All). |
| Self-rated health status | Polytomous / Binary | Contemporaneous | Description of their overall health, assessed as Excellent, Very Good, Good, Fair, or Poor. Fair and Poor were combined because the Poor category had low representation (<5% across all time-points). A binary variable (Excellent/Very good/Good vs. Fair/Poor) was used in the trajectory analyses to simplify interpretation. |
| COVID-19 vaccine status | Polytomous | Contemporaneous | Coded as Fully vaccinated vs. Single dose vs. No vaccine. Fully vaccinated individuals consisted of a single dose of Johnson & Johnson’s vaccine or a double dose of all others. |
| Perceived risk of COVID-19 | Polytomous | Contemporaneous | Perceived risk of acquiring COVID-19 relative to peers. Response categories included Lower, Same, and Higher risk than others like them. |
| Ever had any COVID-19 symptom | Binary | Cumulative prevalence | Ever experiencing COVID-19 symptoms, defined as fever, shortness of breath, sore throat, body aches, fatigue, runny nose or congestion, diarrhea, chills, muscle pain, headache, or a loss of taste or smell. We measured this factor using cumulative prevalence because the T1 question was framed as "ever," while follow-up assessments used a recall period of "in the last 3 months". |
| ***Psychosocial domain*** | | | |
| Loneliness | Polytomous | Contemporaneous | Compared with 3 months ago, felt Less lonely vs. Same Amount of loneliness vs. Lonelier. |
| Social support | Polytomous / Binary | Contemporaneous | Feel they have a network of social support in the place where they live, coded as Yes vs. Don’t know vs. No. In the trajectory analyses, a binary coding was analyzed (Yes vs. Don’t know/No) to simplify interpretation. |
| Psychological distress score | Discrete (Range: 0-24) | Contemporaneous | Created with the Kessler-6, which measures feelings of nervousness, hopelessness, restlessness, sadness, lethargy, and worthlessness over the last 30 days using a 0-4 scale to capture frequency (0 = None, 1 = A little, 2 = Some, 3 = Most, and 4 = All of the time). Respondents with missing data for any measure had their scale value set to missing. In this sample, the Cronbach Alpha for the Kessler-6 scale was 0.84. |
| Moderate-Severe psychological distress | Binary | Contemporaneous | Binary classification of the psychological distress score (Range: 0-24) to capture indications of mild/moderate (scores: 8-12) or severe (scores 13-24) distress vs. no/low (scores: 0-7) distress.   Of those with missing values for the psychological distress scale, we compared the scale values relative to the number of missing data points. If respondents with missing data had a score >8 with their existing data, we coded them as moderate-severe. We coded individuals to the reference category if they were only missing one Kessler-6 measure and their scale score was <4; we used this approach because even if the missing measure was the maximum value (i.e., 4), they would still be classified as no/low. |

Unless specified, Don’t know responses and Non-responses were coded as missing.
