# Supplementary material for: Predictors, patterns, and correlates of moderate-severe psychological distress among New York City College Students during Waves 2–4 of COVID-19
Source: Sci Rep. 2025 Jan 25;15:3206. doi: 10.1038/s41598-025-86364-6 (PMC11762720; doi:10.1038/s41598-025-86364-6)
Supplement: Supplementary file 2 — Supplementary Information 2. [file 41598_2025_86364_MOESM2_ESM.pptx]

## Slide 1
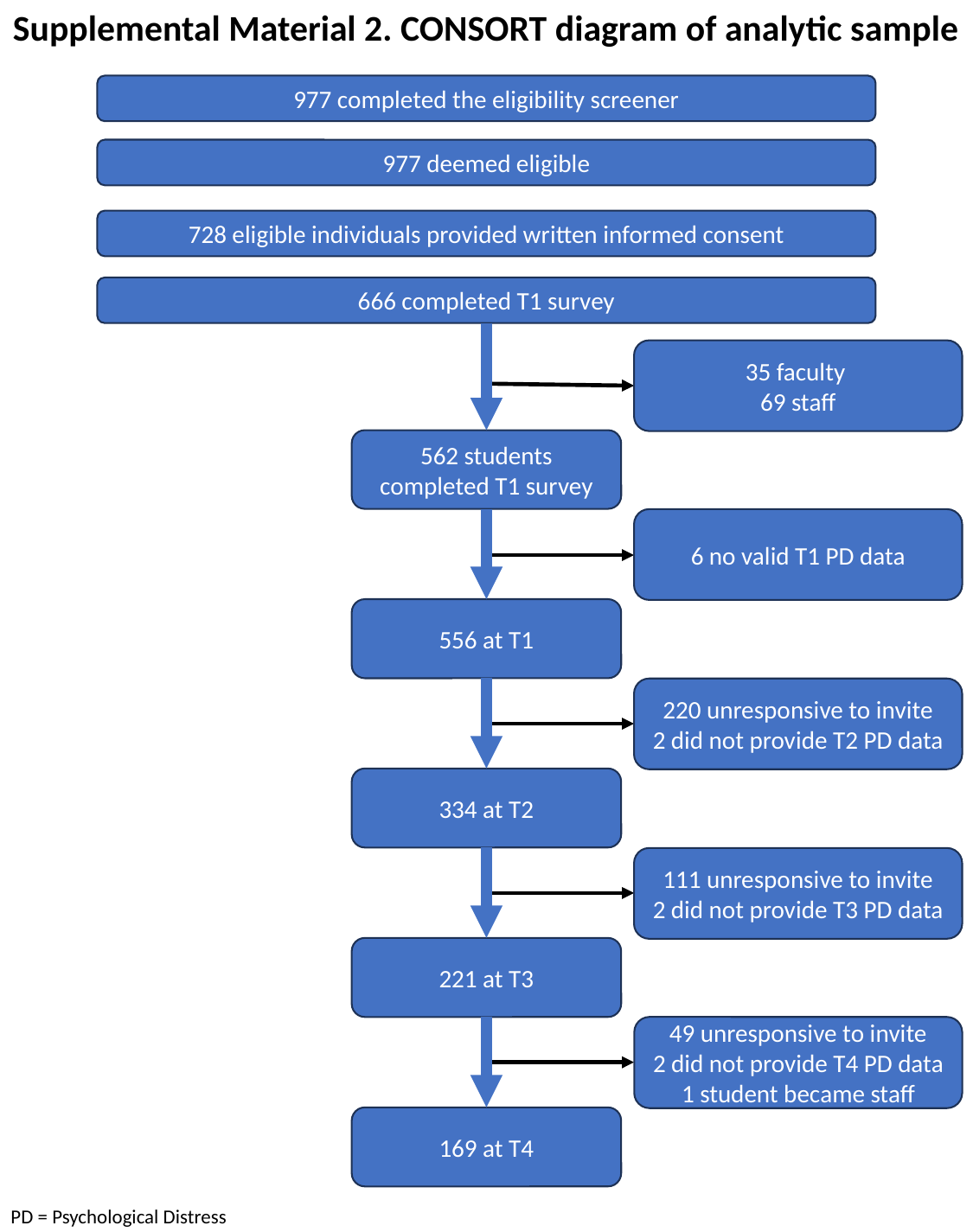

Supplemental Material 2. CONSORT diagram of analytic sample
977 completed the eligibility screener
977 deemed eligible
728 eligible individuals provided written informed consent
666 completed T1 survey
35 faculty
69 staff
562 students completed T1 survey
6 no valid T1 PD data
556 at T1
220 unresponsive to invite
2 did not provide T2 PD data
334 at T2
111 unresponsive to invite
2 did not provide T3 PD data
221 at T3
49 unresponsive to invite
2 did not provide T4 PD data
1 student became staff
169 at T4
PD = Psychological Distress
